# Diagnostics and treatment of female ADHD in the perinatal period

**DOI:** 10.1192/j.eurpsy.2025.263

**Published:** 2025-08-26

**Authors:** A. Todzia-Kornaś

**Affiliations:** Military Institute of Medicine, Warsaw, Poland

## Abstract

**Abstract:**

Pregnancy-related hormonal fluctuations, such as changes in estrogen and progesterone, can exacerbate ADHD symptoms, complicating the diagnostic process. Overlap with symptoms of pregnancy-related conditions, such as fatigue and mood instability, further obscures ADHD identification.

Non-pharmacological interventions, including cognitive-behavioral therapy (CBT) and psychoeducation, are first-line recommendations. For patients requiring pharmacological treatment, stimulant and non-stimulant medications must be considered cautiously, weighing risks such as low birth weight or preterm labor against the potential impact of untreated ADHD on maternal functioning. Emerging data suggest that atomoxetine and certain stimulants may be relatively safe under close monitoring.

Untreated ADHD in pregnant women is associated with higher risks of prenatal stress, inadequate prenatal care, and postpartum depression, highlighting the need for tailored management strategies.

**Keywords:**

ADHD, pregnancy, diagnosis, treatment, pharmacological safety, maternal mental health.

**Disclosure of Interest:**

None Declared

